# Single‐cell transcriptomes of mouse bladder urothelium uncover novel cell type markers and urothelial differentiation characteristics

**DOI:** 10.1111/cpr.13007

**Published:** 2021-02-03

**Authors:** Yan Li, Yaxiao Liu, Zhengdong Gao, Lekai Zhang, Lipeng Chen, Zonglong Wu, Qinggang Liu, Shuai Wang, Nan Zhou, Toby C. Chai, Benkang Shi

**Affiliations:** ^1^ Department of Urology Qilu Hospital Cheeloo College of Medicine Shandong University Jinan China; ^2^ Key Laboratory of Urinary Precision Diagnosis and Treatment in Universities of Shandong Jinan China; ^3^ Laboratory of Basic Medical Sciences Qilu Hospital Cheeloo College of Medicine Shandong University Jinan China; ^4^ Department of Urology Boston University/Boston Medical Center Boston MA USA

**Keywords:** bladder urothelium, cell subtype, single‐cell transcriptome

## Abstract

**Objectives:**

Much of the information to date in terms of subtypes and function of bladder urothelial cells were derived from anatomical location or by the expression of a small number of marker genes. To have a comprehensive map of the cellular anatomy of bladder urothelial cells, we performed single‐cell RNA sequencing to thoroughly characterize mouse bladder urothelium.

**Materials and methods:**

A total of 18,917 single cells from mouse bladder urothelium were analysed by unbiased single‐cell RNA sequencing. The expression of the novel cell marker was confirmed by immunofluorescence using urinary tract infection models.

**Results:**

Unsupervised clustering analysis identified 8 transcriptionally distinct cell subpopulations from mouse bladder urothelial cells. We discovered a novel type of bladder urothelial cells marked by Plxna4 that may be involved with host response and wound healing. We also found a group of basal‐like cells labelled by ASPM that could be the progenitor cells of adult bladder urothelium. ASPM^+^ urothelial cells are significantly increased after injury by UPEC. In addition, specific transcription factors were found to be associated with urothelial cell differentiation. At the last, a number of interstitial cystitis/bladder pain syndrome–regulating genes were found differentially expressed among different urothelial cell subpopulations.

**Conclusions:**

Our study provides a comprehensive characterization of bladder urothelial cells, which is fundamental to understanding the biology of bladder urothelium and associated bladder disease.

## Graphical abstract

Much of the information to date in terms of subtypes and function of bladder urothelial cells were derived from anatomical location or by the expression of a small number of marker genes. To have a comprehensive map of the cellular anatomy of bladder urothelial cells, we performed single‐cell RNA sequencing to thoroughly characterize mouse bladder urothelium. A total of 18 917 single cells from mouse bladder urothelium were analysed by unbiased single‐cell RNA sequencing. The expression of the novel cell marker was confirmed by immunofluorescence using urinary tract infection models. Unsupervised clustering analysis identified 8 transcriptionally distinct cell subpopulations from mouse bladder urothelial cells. We discovered a novel type of bladder urothelial cells marked by Plxna4 that may be involved with host response and wound healing. We also found a group of basal‐like cells labelled by ASPM that could be the progenitor cells of adult bladder urothelium. ASPM^+^ urothelial cells are significantly increased after injury by UPEC. In addition, specific transcription factors were found to be associated with urothelial cell differentiation. At the last, a number of interstitial cystitis/bladder pain syndrome–regulating genes were found differentially expressed among different urothelial cell subpopulations. Our study provides a comprehensive characterization of bladder urothelial cells, which is fundamental to understanding the biology of bladder urothelium and associated bladder disease.

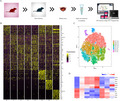

## 1 INTRODUCTION

The bladder urothelium is a stratified epithelium that serves as a crucial barrier between the blood and urine. Beside barrier function, the bladder urothelium also has a range of other important functions such as sensory function and immune function.^1^, ^2^ The bladder urothelium plays an important role by actively communicating with bladder nerves, smooth muscle cells and cells belonging to the immune systems, which make bladder urothelium as a sophisticated communication system. Anatomically, the bladder urothelium consists of a layer of basal cells, intermediate cells and a luminal layer of fully differentiated superficial cells. The apical layer of urothelial cells is considered as the prime contributor to urothelial impermeability function. Cell progenitors in the basal and intermediate layer are responsible for the urothelial regeneration in response to injury or infection.^3^, ^4^ Normal bladder urothelial function is critical in maintaining the homeostasis of bladder. Many bladder diseases, in particular, the bladder cancer, bladder pain syndrome/interstitial cystitis (BPS/IC) and bacterial cystitis are related to bladder urothelial biology and function. Investigating the bladder urothelial cell types, specific cell markers, signalling receptors and genes will help us to learn more about the relationship between bladder urothelial cells and bladder diseases including BPS/IC, bladder cancer and urinary tract infections (UTIs).

Since the pathophysiology and treatment strategies of bladder diseases are highly relevant to the bladder urothelium, it is vital to understand the biology and physiology of bladder urothelium. Much of the information to date in terms of subtypes and function of bladder urothelial cells were derived from anatomical location or by the expression of a small number of marker genes. Common technique to study the bladder urothelium is using cell culture preparations and enzyme digested or surgically isolated tissue.^5^, ^6^ However, it seems difficult, or even impossible, to study cell type–specific characteristics and functions of bladder urothelial cells by these traditional methods. The heterogeneity and hierarchy of bladder urothelial cells have not been well revealed.

Single‐cell RNA sequencing is an advanced technology that can analyse mRNA transcripts from thousands of individual cells simultaneously, and so greatly facilitates the studies on cell function under normal physiological activities as well as during disease processes.^7^ By utilizing this powerful tool, the cellular hierarchy and developmental trajectory of multiple organs have been discovered, such as lung, kidney and intestine.^8^, ^9^, ^10^ Although previous studies have mapped all major mouse organs, including mouse bladder,^11^, ^12^ high‐throughput single‐cell RNA sequencing of bladder urothelial cells have not been reported so far. The present study aims to investigate the unsupervised classification and function of mouse bladder urothelial cells precisely by single‐cell RNA sequencing. The transcriptomic map of bladder urothelial cells will provide a resource for studying bladder urothelial cell types, specific cell markers, signalling receptors and genes that can help us to learn more about the relationship between bladder urothelial cells and bladder diseases.

## 2 MATERIALS AND METHODS

### 2.1 Bladder urothelial cell isolation

Ten‐week‐old female C57BL/6 mice were ordered from the animal experimental centre of Shandong University. All mice were maintained in the specific pathogen‐free animal facility of our institution. Five mice were sacrificed by cervical vertebra dislocation and bladder was removed. The bladder was cut open through the bladder neck with sharp scissor and was nailed to the Sylgard‐coated plate (Supplement Figure S1). The lumen of the bladder was exposed using four pins to form a parallelogram. The bladder was incubated in dispase II solution (04 942 078 001, Sigma) at 37°C for 1.5 hours. The bladder was then enlarged to achieve a maximum stretch (Supplement Figure S1E). After dispase II solution was discarded, 10‐15ml PBS‐EDTA (10mmol/L, CZ0026, Leagene) was added to Sylgard‐coated plate and shaking for 1.5 hours. The PBS‐EDTA was collected and centrifuged at 300g for 5 minutes. The supernatant was aspirated and the cells were then digested in 1ml TrypLE™ Express Enzyme (12604‐013, Thermo Fisher Scientific) for 1min. 10ml 0.04% BSA D‐PBS solution (D1040, Solarbio) was added to stop the digestion and then centrifuged at 300g for 5 minutes. Cells were then resuspended in 10ml 0.04% BSA D‐PBS solution and were sequentially filtered through 70 and 40 μm filters. We obtained the single‐cell suspension and detected live cells using a haemocytometer with 0.4% trypan blue solution (C0040, Solarbio). The viability percentage was calculated by Calcein‐AM/PI staining. Approval was obtained from the Laboratory Animal Ethical and Welfare Committee of Shandong University Cheeloo College of Medicine (NO. 19 062).

### 2.2 Murine UTI model

Female C57BL/6 mice (10 weeks old) were anaesthetized, and cystitis was induced by transurethral inoculation of 100 µl of a uropathogenic Escherichia coli (CF073) suspension in PBS (1‐2 × 10^7^ colony‐forming units) via a catheter designed for mice (inner diameter 0.28 mm) as previously described.^13^

### 2.3 Single‐cell RNA sequencing

Single‐cell RNA‐seq libraries were prepared with Chromium Single cell 3’ Reagent (v3) Kits according to the manufacturer's protocol. Single‐cell suspensions were loaded on the Chromium Single Cell Controller Instrument (10 × Genomics) to generate single‐cell gel beads in emulsions (GEMs). Briefly, 10^5^‐10^6^ single cells were suspended in calcium‐ and magnesium‐free PBS containing 0.04% weight/volume BSA. After generation of GEMs, reverse transcription reactions were engaged barcoded full‐length cDNA followed by the disruption of emulsions using the recovery agent and cDNA clean up with DynaBeads Myone Silane Beads (Thermo Fisher Scientific). cDNA was then amplified by PCR with appropriate cycles which depend on the recovery cells. Subsequently, the amplified cDNA was fragmented, end‐repaired, A‐tailed, index adaptor ligated and library amplification. Then, these libraries were sequenced on the Illumina sequencing platform (HiSeq X Ten) and 150 bp paired‐end reads were generated. The single‐cell RNA‐seq data are available at GEO: GSE163029.

### 2.4 Quality control

The Cell Ranger software pipeline (version 3.0.0) provided by 10 × Genomics was used to demultiplex cellular barcodes, map reads to the genome and transcriptome using the STAR aligner and down‐sample reads as required to generate normalized aggregate data across samples, producing a matrix of gene counts versus cells. We processed the unique molecular identifier (UMI) count matrix using the Seurat R package (version 2.3.4; https://satijalab.org/seurat/). To remove low‐quality cells and likely multiplet captures, which is a major concern in microdroplet‐based experiments, we apply a criterion to filter out cells with UMI/gene numbers out of the limit of mean value ± 2‐fold of standard deviations assuming a Guassian distribution of each cells' UMI/gene numbers. Following visual inspection of the distribution of cells by the fraction of mitochondrial genes expressed, we further discarded low‐quality cells where > 10% of the counts belonged to mitochondrial genes. After applying these QC criteria, 18,917 single cells and 31,053 genes in total remained and were included in downstream analyses. Library size normalization was performed in Seurat on the filtered matrix to obtain the normalized count.

### 2.5 Dimensional reduction, clustering and cell type identification

Top variable genes across single cells were identified using the method as previously described.^7^ Briefly, the average expression and dispersion were calculated for each gene, and genes were subsequently placed into 8 bins based on expression. Principal component analysis (PCA) was performed to reduce the dimensionality on the log transformed gene‐barcode matrices of top variable genes. Cells were clustered based on a graph‐based clustering approach and were visualized in 2‐dimension using t‐SNE. Likelihood ratio test that simultaneously test for changes in mean expression and in the percentage of expressed cells was used to identify significantly differentially expressed genes between clusters. Here, we use the R package SingleR, a novel computational method for unbiased cell type recognition of scRNA‐seq, with reference transcriptomic data sets ‘Immgen’ to infer the cell of origin of each of the single cells independently and identify cell types.^14^

### 2.6 RNA velocity analysis

We performed RNA velocity analysis using the R package velocyto.R v0.6.^15^ The RNA velocity was calculated on the basis of spliced and unspliced transcript reads and estimated using gene‐relative model. The resulting velocity estimates were projected onto the t‐SNE embedding obtained in Seurat.

### 2.7 SCENIC analysis

We used SCENIC (Single Cell rEgulatory Network Inference and Clustering) for the simultaneous reconstruction of gene regulatory networks (GRNs) and the identification of stable cell states.^16^ SCENIC contains three main steps, including co‐expression analysis, target gene motif enrichment analysis and regulon activity evaluation. The main outcomes contain a list of regulons (each representing a transcription factor along with a set of co‐expressed and motif significantly enriched target genes), and the regulon activity scores for each cell. In detail, SCENIC analysis was run using the motif database for RcisTarget and GRNboost (SCENIC version 1.1.2.2, which corresponds to RcisTarget 1.2.1 and AUCell 1.4.1) with default parameters. We identified transcription factor binding motifs over‐represented on a gene list with RcisTarget package. The activity of each group of regulons in each cell was scored by AUCell package. To evaluate the specific regulon for each cell type, we calculated the regulon specificity score which is based on the Jensen‐Shannon divergence, a measure of the similarity between two probability distributions. To systematically characterize the combinatorial patterns, we compared the atlas‐wide similarity of regulon activity scores of every regulon pair based on the Connection Specificity Index (CSI).^17^, ^18^, ^19^ The CSI for all regulons was calculated with scFunctions package.

### 2.8 Pseudotime analysis

We determined the developmental pseudotime with the Monocle2 package.^20^ The data, previously scaled and clustered by the Seurat tool, were loaded into a monocle object with default parameters. We obtained variable genes with Monocle2 and ordered the cells onto a pseudotime trajectory based on the union of highly variable genes obtained from all cells.

### 2.9 HE stain and immunofluorescence

The mice bladder tissues were kept in 10% formalin and embedded in paraffin. Paraffin sections were stained for haematoxylin and eosin. The bladder sections were incubated overnight at 4°C with primary Anti‐Plexin A4 (1:200; Abcam, cat. no. ab39350) and Anti‐ASPM (1:200; NOVUS, catalog number: NB100‐2278), respectively. After washing with PBS, the slides were incubated with secondary Alexa Fluor 488 (goat anti‐Rabbit IgG preadsorbed, 1:500, ab150081, Abcam) for 1 hour. After washing with PBS, the slides were stained with DAPI (ab104139, Abcam) for 10 minutes.

## 3 RESULTS

### 3.1 Single‐cell profiling and unbiased clustering of mouse bladder urothelial cells

**FIGURE 1:**
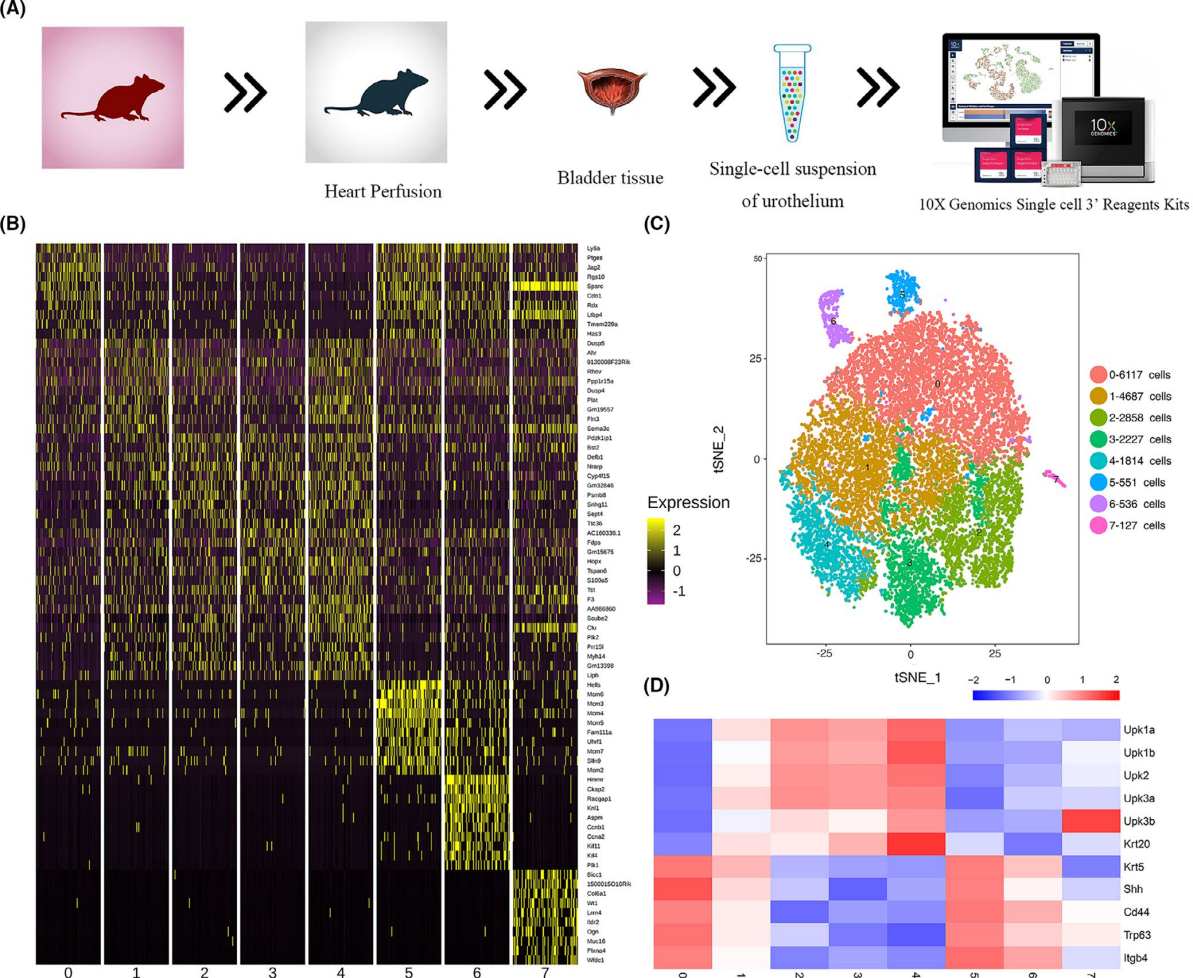
Cell diversity of mouse urinary bladder urothelial cells delineated by single‐cell transcriptomic analysis (A) schematics of the experimental design for single‐cell RNA sequencing. The mouse bladder urothelum were collected and processed into a single‐cell suspension. Single‐cell RNA sequencing was performed using the 10x‐Based Genomics platform. B, Heat map showing the differential genes distinguishing the cell subtypes by using Seurat tool. C, Unsupervised clustering demonstrates 8 subtypes shown in a t‐SNE 2D map. (D) Heat map showing the expression of typical urothelial cell type–specific markers genes in each cluster

Single bladder urothelial cell suspension was obtained from the mouse bladder urothelium using trypsin digestion (Supplement Figure S1). Trypan blue staining and Calcein‐AM/PI staining showed that the per cent of alive cell is more than 90% (Supplement Figure S2). Mouse bladder urothelial cell types were catalogued in an unbiased manner using droplet‐based single‐cell RNA sequencing (Figure 1A). Using stringent quality controls (Supplement Figure S3), 18 917 bladder urothelial cells were further analysed. Unsupervised clustering analysis identified 8 distinct cell clusters consisting of as few as 127 cells to as many as 6117 cells per cluster (the clusters were censored for a minimum of 100 cells) (Figure 1B and 1C).

### 3.2 Classification of urothelial cells based on cell type–specific marker genes

**FIGURE 2:**
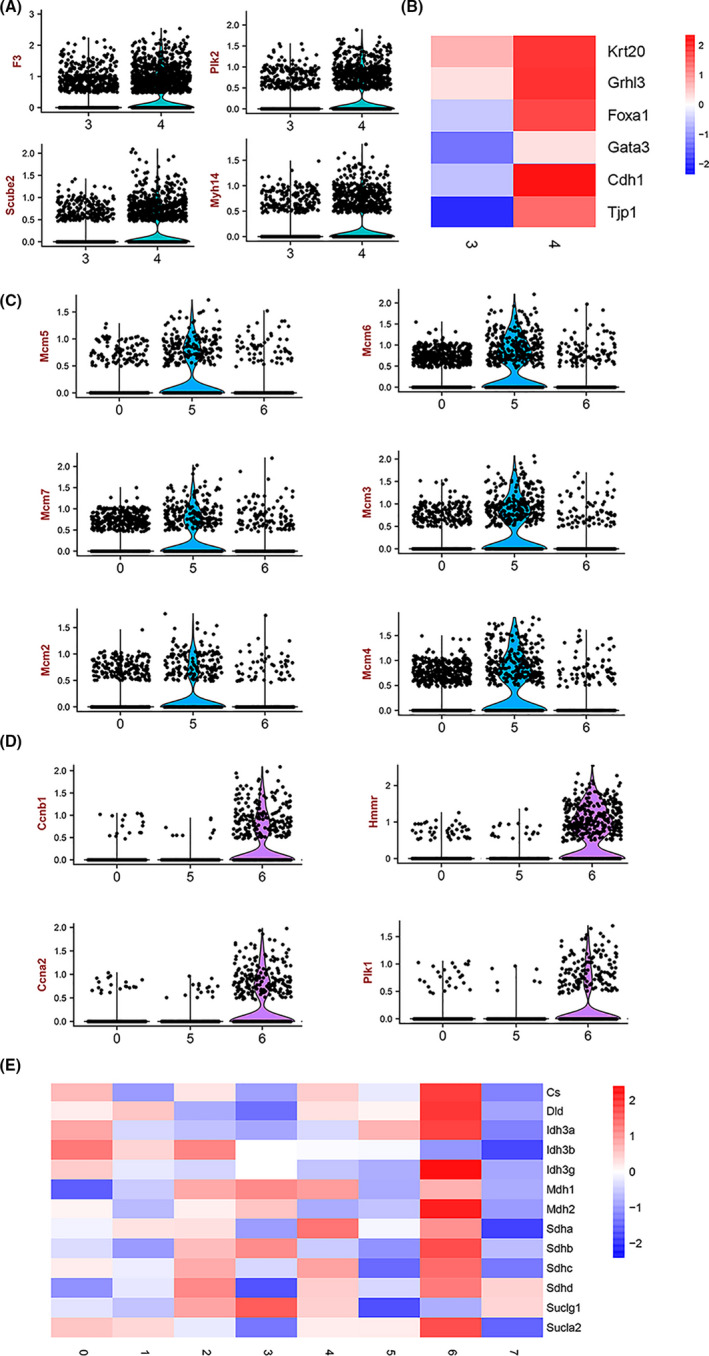
Heterogeneity of bladder urothelial cell subtypes based on marker genes. A, Violin plot showing the main differential genes (F3, PIK2, Scube2 and Myh14) between cluster 3 and cluster 4. B, Heat map showing the expression of tight junctions–associated genes (CDH1 and Tjp1) and cell differentiation‐associated genes (Foxa1, Gata3 and Grhl3) in cluster 3 and cluster 4. C, Violin plot showing the cluster 5 was highly enriched for DNA helicase gene markers. D, Violin plot showing the cluster 6 was highly enriched cell cycling regulatory gene. (E) Heat map showing that cluster 6 was enriched for tricarboxylic acid (TCA) cycle regulating genes, suggesting those cell subtypes possess active metabolism function

To further investigate the identity of each cell cluster, we performed differential gene expression analysis (Figure 1D). According to previous studies,^4^, ^21^, ^22^, ^23^, ^24^ the superficial cells were marked by the expression of keratin‐20 and uroplakin (Upk) gene family (UpkIa, Ib, П, Шa and Шb). The basal cells were marked by keratin‐5, integrin β4 (ITGB4), sonic hedgehog (Shh) and transcription factor p63 (Trp63) and exhibit undetectable expression of keratin‐20 or Upk. The intermediate cells are marked by Upk and display undetectable expression of keratin‐20 or keratin‐5. Based on the specific expression of these known cell type–specific markers, cluster 0 was identified as basal‐like cells, which was named as basal cell 1 (B1). Cluster 1 was positive for both superficial cell markers (Upk Ia, Upk IIIb) and basal cell maker (keratin‐5). These data raise the hypothesis that there might be a transitional stage between basal cells and superficial cells. Hence, cluster 1 was named as basal to superficial cells (BTS). Cluster 2 was marked by Upk and display undetectable expression of keratin‐20 or keratin‐5. It is named as intermediate cell. Cluster 3 and cluster 4 were defined as superficial cell 1 and superficial cell 2 (S1 and S2). Although both cluster 3 and cluster 4 were defined as superficial cells, there was difference in the expression of some marker genes including F3, Plk2, Scube2 and Myh14 (Figure 2A). Cluster 4 was highly enriched for CDH1 and Tjp1, which form scaffolding in tight junctions^25^ (Figure 2B). In addition, cluster 4 has higher expression of genes including Foxa1, Gata3 and Grhl3, which are typically associated with differentiated urothelial cell phenotypes.^21^, ^23^ To further investigate the differences between these two clusters, we identified their differential genes. Five genes (Ivl, c‐Jun, Klf6, Psca and Rab11fip1) increased ≥ 2‐fold change in cluster 4 and two genes increased ≥ 2‐fold change in cluster 3 (Supplement Figure S4). Cluster 5 and cluster 6 were classified into basal cell category because of expression of keratin‐5, ITGb4, Trp63 and Shh. In addition, cluster 5 was enriched for DNA helicase gene markers (Figure 2C) and cluster 6 was enriched for cell cycling regulatory gene (Figure 2D). Further analysis also showed that was enriched for tricarboxylic acid (TCA) cycle regulating genes, suggesting those cell subtypes possess active metabolism function (Figure 2E). Cluster 5 and cluster 6 were named B2 and B3, respectively. Cluster 7 was not enriched with any of the known subtype‐specific markers except for Upk IIIb. Cluster 7 was named as Plxna4^+^ urothelial cell according to the most specific gene—Plxna4 (Figure 2E). Altogether, the present single‐cell transcriptome atlas provides a molecular definition of 8 bladder urothelial cell types.

### 3.3 Reconstructing the developmental trajectory of mouse bladder urothelial cells

**FIGURE 3:**
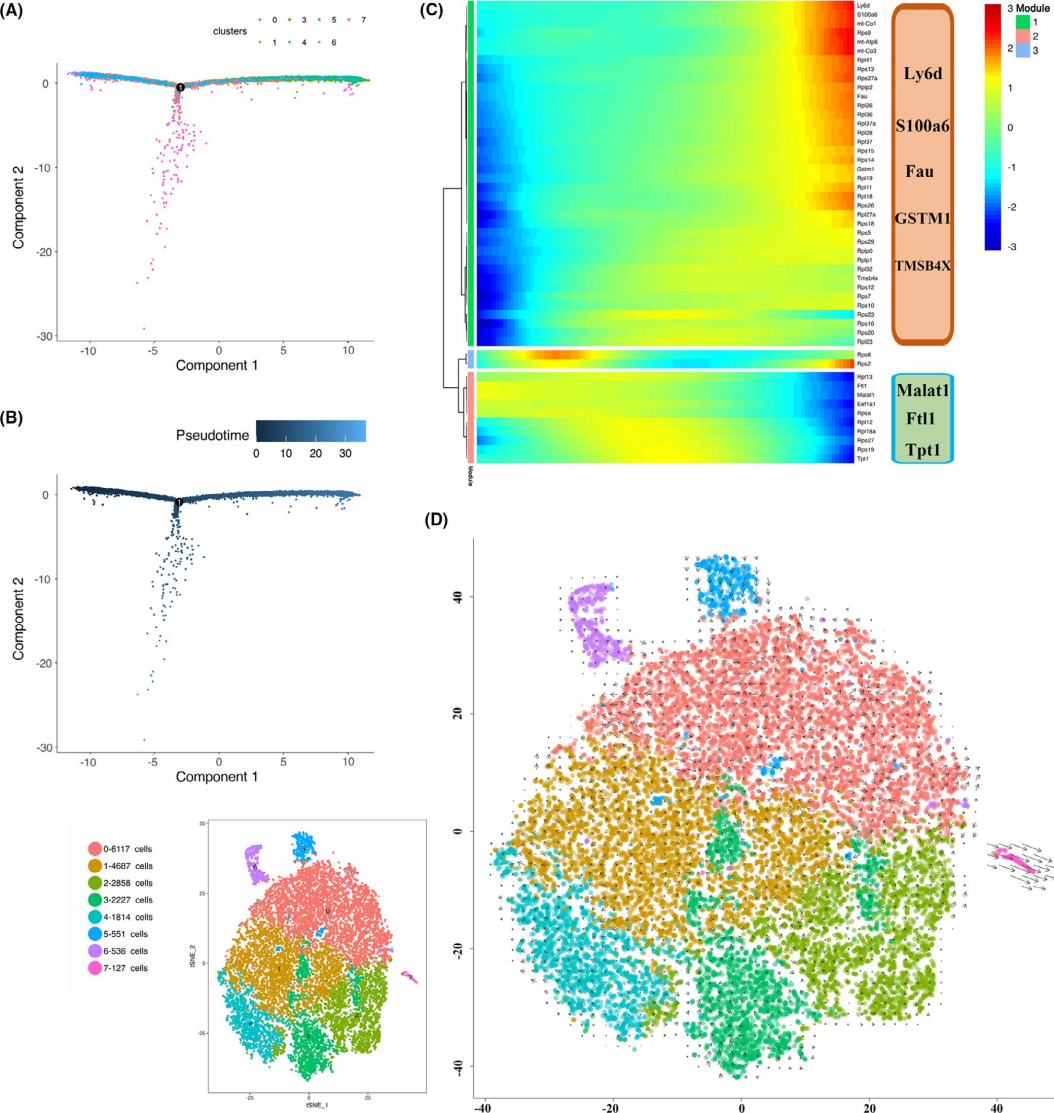
Reconstructing the developmental trajectory and the pseudotime analysis of bladder urothelial cells. A, Monocle2‐generated pseudotemporal trajectory of 8 urothelial cell subtypes imported from Seurat data, colored by cell‐name designation. B, Pseudotime was colored in a gradient from dark to light blue, and the start of pseudotime was indicated. C, Heat map for clustering the top 50 genes that vary as a function of pseudotime. The 50 genes were divided into three clusters (cluster 1, cluster 2 and cluster 3), representing the genes at the beginning stage, the transitory stage and the end stage of developmental trajectory, respectively. D, Pseudotime analysis of urothelial cells by using RNA velocity reconstructed the developmental lineages

It is usually believed basal cells can divide and produce superficial cell daughters. To further understand the relationship among different types of bladder urothelial cells, we utilized reverse graph embedding to generate a trajectory plot (Figure 3A) and perform cell trajectory analysis by using pseudotime analysis (Figure 3B). Cluster 6 is the starting point of the trajectory, and followed by cluster 5 and cluster 0. BTS cells were in the middle position which suggested its transitional state. Superficial cell (cluster 3 and cluster 4) and Plxna4 + urothelial cells appeared as the two ends of the trajectory branch 1. It suggested cluster 6 might be the progenitor cell and Plxna4^+^ urothelial cell might play as a superficial cell. To further analyse the genes in terms of the changes in pseudotime analysis, we clustered genes via a pseudotemporal expression pattern. The top 50 genes that vary as a function of pseudotime were clustered, as shown by the heat map (Figure 3C). We found that Malat1, Ftl1 and Tpt1 were highly expressed at the early stage of developmental trajectory, while Ly6d, S100a6, Fau, Gstm1 and Tmsb4x were highly expressed at the end stage of developmental trajectory. These genes were suggested to regulate cellular pluripotency or identified as growth inducible genes and might be linked to the process of urothelial cell proliferation and differentiation.

### 3.4 RNA velocity analysis revealed the developmental lineages

RNA velocity was analysed to study the developmental lineage of urothelial cells. The direction of the vector reflects the direction of cell lineage development, and the length of the vector reflects the rate. RNA velocity projected on the t‐SNE revealed the developmental lineages of bladder urothelial cells (Figure 3D). It was showed that B3 cell (cluster 6) was responsible for the development of B2 cell (cluster 5) and B2 cell was involved in the development of B1 cell (cluster 0). BTS (cluster 1) and intermediate cell (cluster 2) were involved in the development of S1 and S2 (cluster 3 and cluster 4).

### 3.5 Identification of a novel Plxna4 + bladder urothelial cell type

**FIGURE 4:**
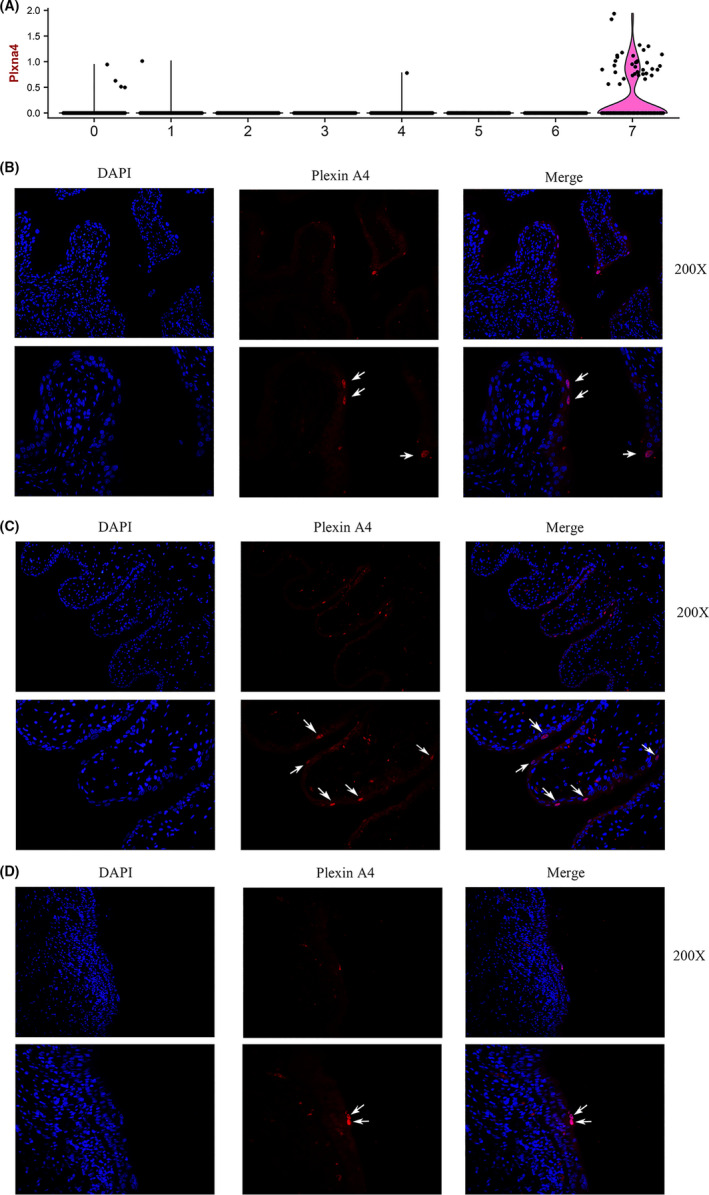
Identification of Plxan4 + cells in bladder tissues. A, Violin plot showing the expression of Plxan4 in each cluster. B, IF analysis of the expression of Plxan4 (Red) and DNA staining by DAPI (Blue) within the tissue paraffin sections from mouse bladder. C, IF analysis of the expression of Plxan4 in rat bladder. (D) IF analysis of the expression of Plxan4 in human bladder

Cluster 7 was enriched for Plxna4 (Figure 4A) and named as Plxna4^+^ urothelial cell. Plxna4^+^ urothelial cells belong to the superficial cells since Upk Шb was highly expressed in this cluster. Interestingly, Plxna4^+^ urothelial cells were negative for Keratin‐20, which is a urothelial differentiation marker expressed in superficial cells. These results indicated that Plxna4^+^ urothelial cell group is a special cell type of bladder urothelial cells. Further analysis showed Plxna4^+^ urothelial cells were also enriched for a range of genes including WFDC1, BICC1, COL6A1 and WT1, OGN. Since Plxna4^+^ urothelial cells have never been reported before, we further investigate the expression of Plxna4 in the bladder urothelium of mouse, rat and human beings. Immunofluorescence results showed that a group of superficial cells in the mouse bladder urothelium were positive for Plxna4 (Figure 4B). Especially, Plxna4^+^ bladder urothelial cells were located in the outermost layer of bladder urothelium. Similar results were also obtained in the bladder urothelium of rats and human beings (Figure 4C and 4D).

### 3.6 Identification of an ASPM + basal cell type

**FIGURE 5:**
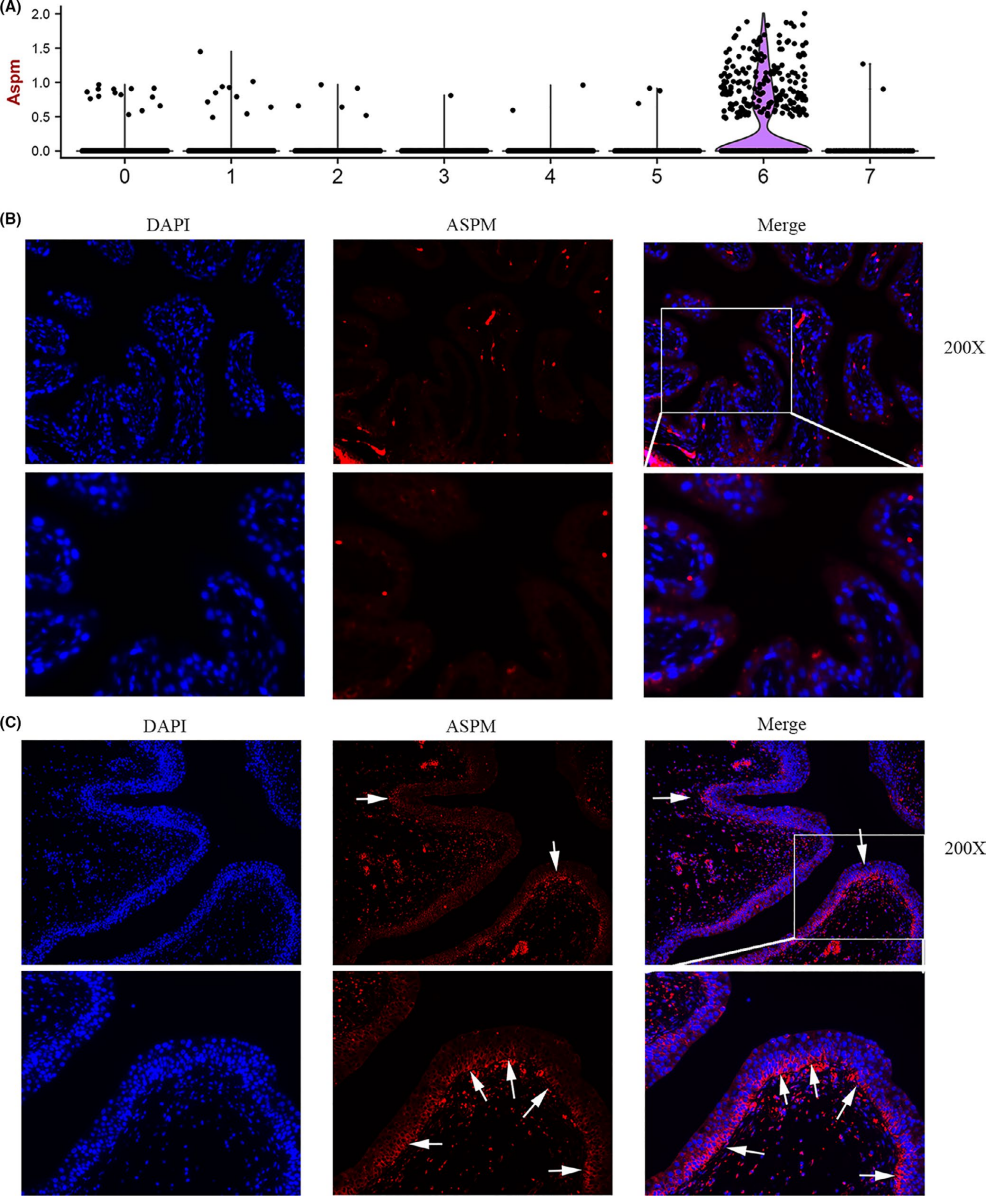
Elevated expression of ASPM + basal cells was found in the response to UPEC injury. A, Violin plot showing the expression of ASPM in each cluster. B, IF analysis of the expression of ASPM (Red) and DNA staining by DAPI (Blue) within the tissue paraffin sections from mouse bladder tissue. C, IF analysis of the expression of ASPM in bladder tissue 48h after UPEC infection. ASPM + basal cells were increased in urothelial regeneration stage after UPEC injury

Cluster 6 was distinguishable from the other cell types by the expression of the marker gene Abnormal Spindle Microtubule Assembly (ASPM) (Figure 5A). So we named this group as the ASPM^+^ urothelial cells. Genome‐wide analysis has suggested ASPM as a possible stem/progenitor cell marker.^26^, ^27^ Immunofluorescence results showed that few ASPM^+^ cell was found in healthy mouse urothelium (Figure 5B). However, the numbers of ASPM^+^ basal cells were significantly increases in basal layer after UPEC injury (Figure 5C).

### 3.7 The expression patterns of transcription factors in bladder urothelial cells

**FIGURE 6:**
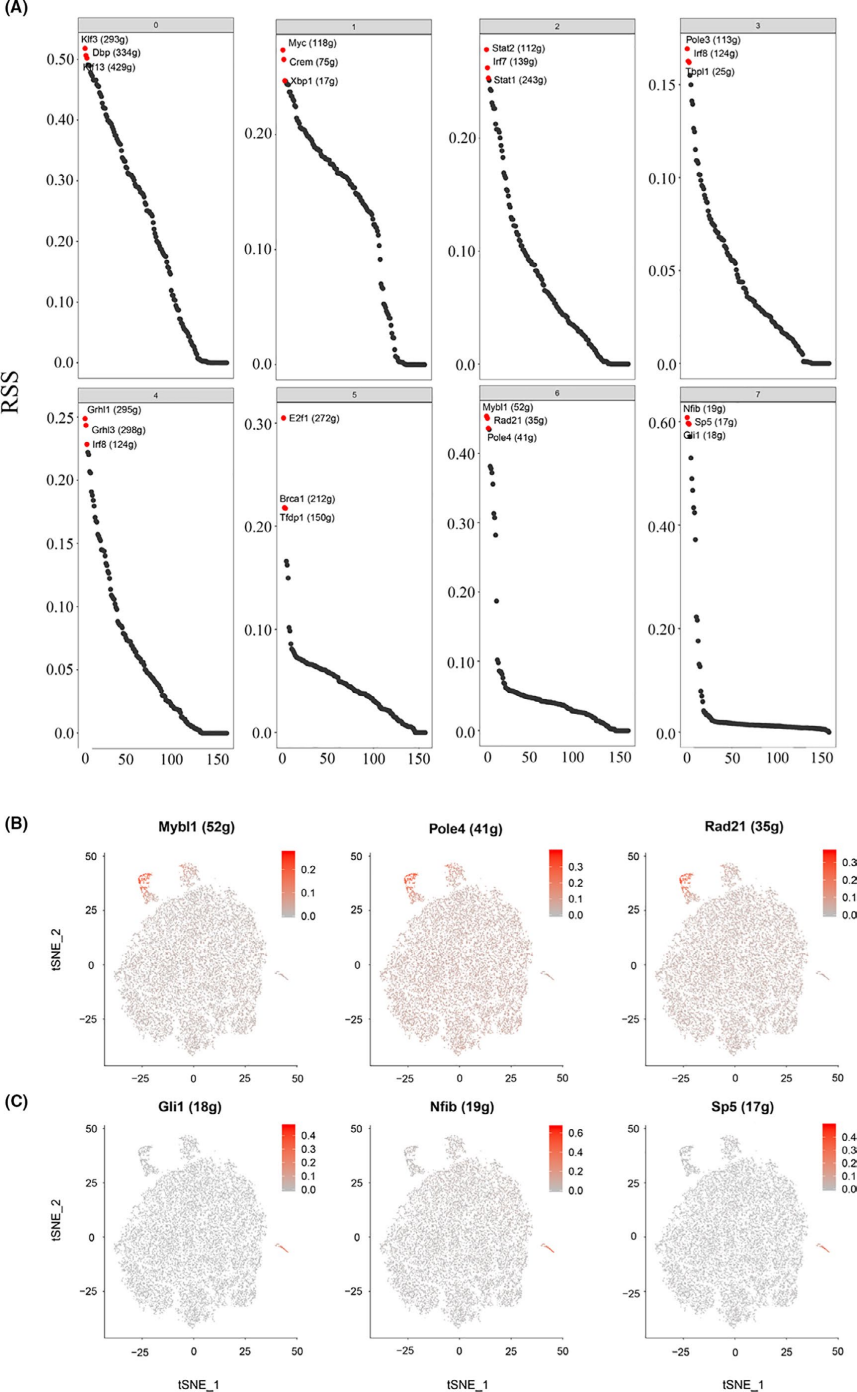
Cell type–specific regulon activity analysis. A, Rank for regulons in all cell types based on regulon specificity score (RSS). B, Binarized regulon activity scores (RAS) for top regulons of Cluster 6 on t‐SNE map, suggesting Mybl1, Rad21 and pole4 could be critical regulators for Cluster 6 identity. C, RAS for top regulons of Cluster 7 on t‐SNE map, suggesting Nfib, sp5 and Gli1 could be critical regulators for cluster 7 identity

The maintenance of cell identity involves the coordinated action of many regulators, among which transcription factors have been long recognized to play a central role.^18^ SCENIC was used to identify transcription factors in different bladder urothelial cell subpopulations and to predict essential regulators for urothelial cell type. Our network analysis identified top 3 specific regulons for each cluster (Figure 6A). Mybl1, Rad21 and pole4 may cooperate in regulating ASPM^+^ cell differentiation. Nfib, sp5 and Gli1 may cooperate in regulating Plxna4^+^ cell differentiation. t‐SNE plot provides additional support that the activities of these regulons are highly specific to ASPM^+^ cell subpopulations (Figure 6B) and Plxna4^+^ cell subpopulations (Figure 6C). This analysis provides an opportunity to systematically identify critical regulators for urothelial cell identity.

**FIGURE 7:**
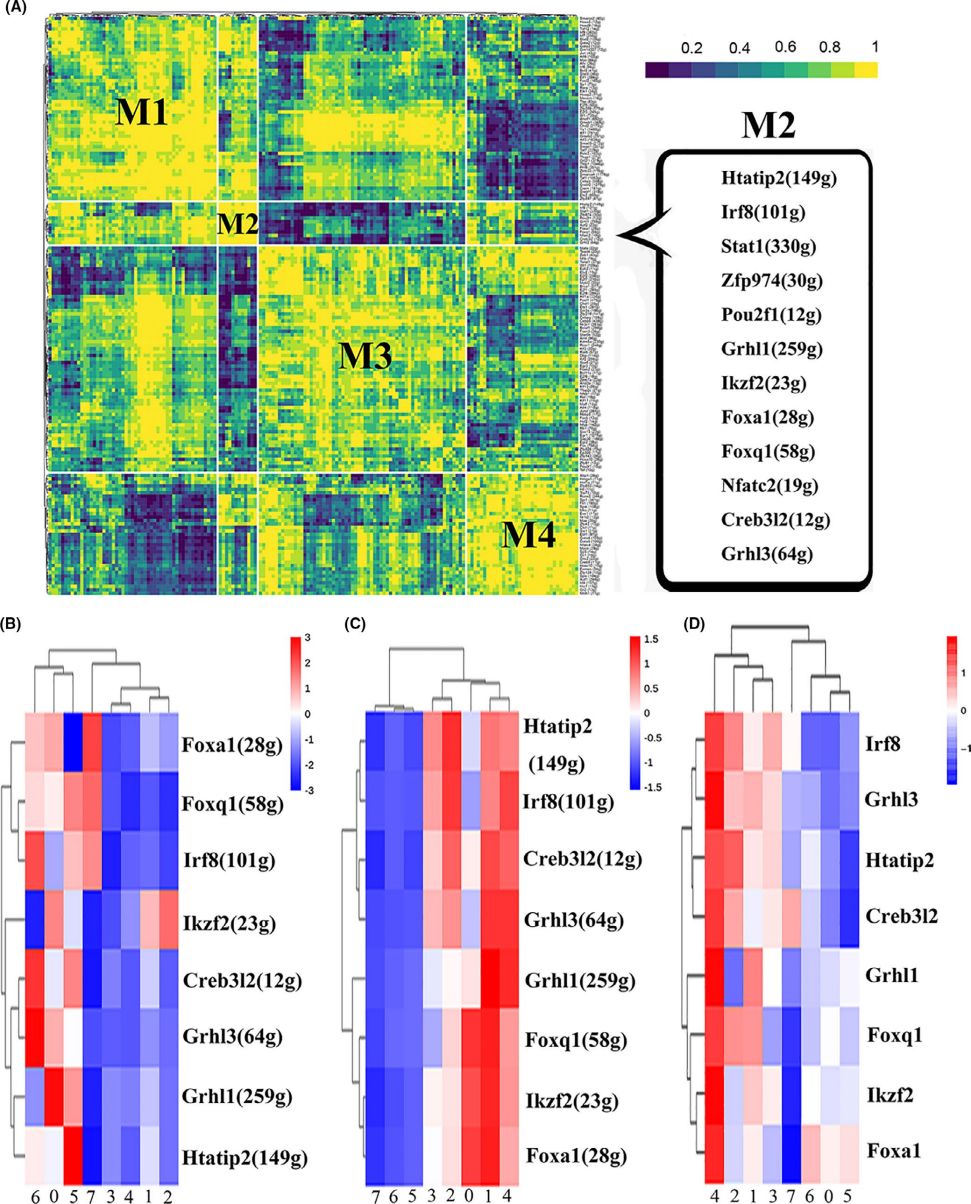
Identification of combinatorial regulon modules (A) Regulons are organized into 4 major modules (M1‐M4) by regulon connection specificity index (CSI) matrix. M2 was identified based on representative transcription factors. B, Heat map shows the regulon activity score of M2 regulons in each cluster. C, Heat map shows the regulon specificity score of M2 regulons in each cluster. D, Heat map shows the expression of transcription factors of M2 regulons in each cluster

Transcription factors often work in combination to coordinate gene expression levels. To systematically characterize the combinatorial patterns, we compared the atlas‐wide similarity of regulon activity scores of every regulon pair based on the CSI. We identified co‐expression modules associated with 165 transcription factors, ranging in size from 10 to 2172 target genes, with a median size of 50 genes. The regulons which have similar functions were categorized into a module. Strikingly, regulons were organized into 4 major modules. The difference of CSI activity in each cluster was not substantial. Module M2 contains 12 regulators, in which Foxa1, Grhl3, Foxq1, Grhl1, Htatip2, Ikzf2, Irf8 and Creb3l2 were considered to play important role in cell differentiation (Figure 7A). The activity of those regulons was higher in basal‐like cell subpopulations (clusters 0, 5 and 6) but lower in S2 cell subpopulations (cluster 4) (Figure 7B). On the contrary, the regulon specificity score (RSS) were higher in S2 cell (cluster 4) and BTS cell (cluster 1) (Figure 7C), which was similar to gene expression level (Figure 7D). It indicated that combinations of these regulons play important roles in urothelial cell differentiation. The RSS and gene expression of transcription factors, rather than activity of regulons, may more accurately reflect its identity. In order to investigate the function of these 8 regulons, their target genes were searched by KEGG pathways database. It was found that the target genes of these 8 regulons are associated with epidermal barrier formation, such as regulation of actin cytoskeleton, bacterial invasion of epithelial cells, endocytosis and lysosome (Supplement Figure S5).

### 3.8 Cell type–specific expression of IC/BPS‐associated genes

**FIGURE 8:**
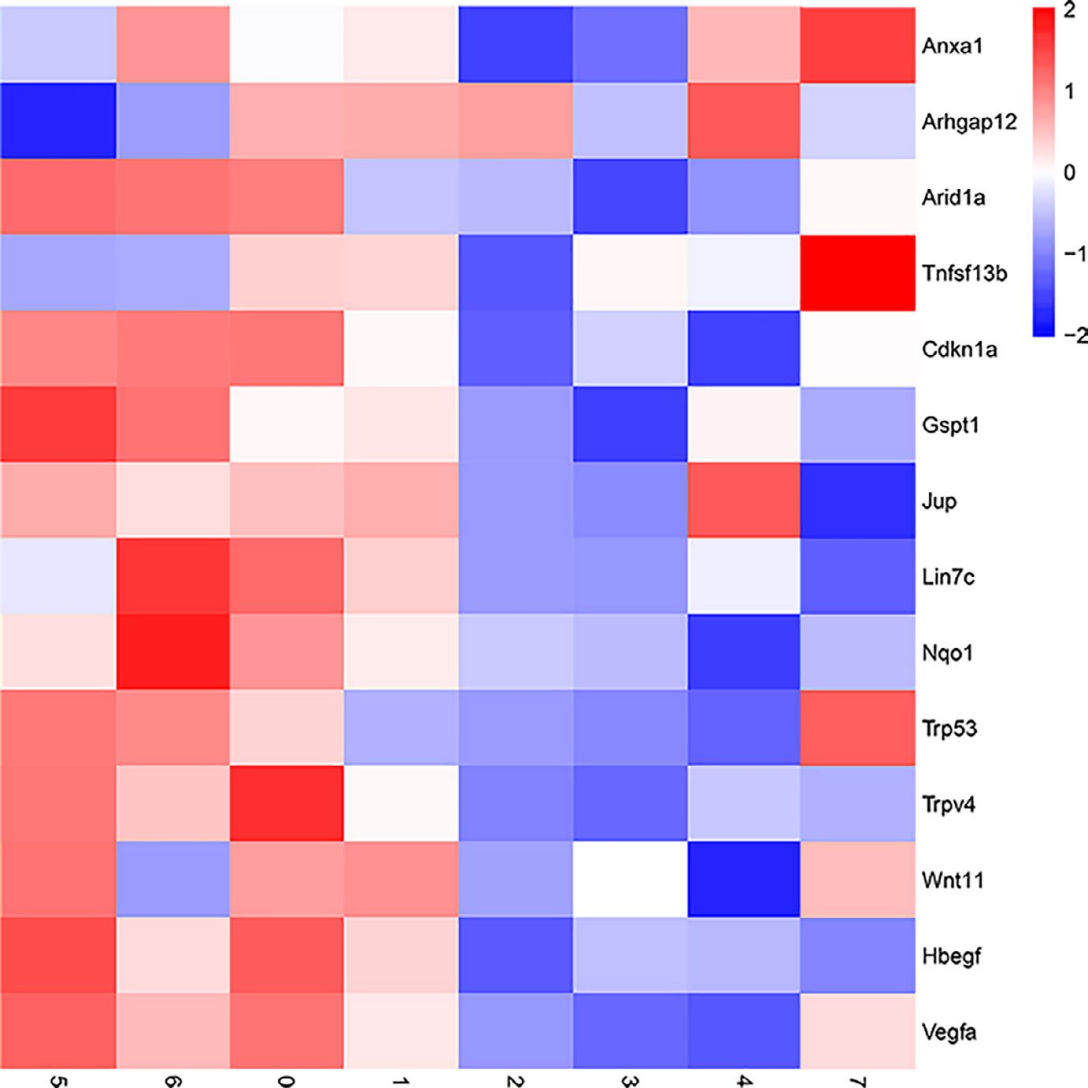
Heat map showing basal cell was enriched for IC/BPS regulating genes

Since the pathogeneses of IC/BPS is theorized to be associated with impairment of the urothelium, we asked whether specific bladder urothelial cell subtypes are enriched for IC/BPS‐associated genes. We investigated the expression profiles of genes known to contribute to pathogeneses of IC/BPS.^28^, ^29^, ^30^ We found a number of IC/BPS‐associated genes differentially expressed among different subtypes (Figure 8). Compared with other subpopulations, basal‐like cell subpopulations (B1, B2 and B3) and BTS subpopulation showed greater expression of these IC/BPS‐associated genes.

## 4 DISCUSSION

Identification of bladder urothelial subtypes and single‐cell transcriptomic map are important for the understanding the biology of bladder as well as the pathogenesis of bladder diseases. However, to our knowledge, previous studies lacked an integrated perspective for studying bladder urothelial cells. The anatomical classification of bladder urothelial cells should be challenged. The number of existing bladder urothelial cell subtypes and their distinctive properties are unclear. By using the single‐cell RNA sequencing techniques, the present study classified mouse bladder urothelial cells into eight subpopulations according to the cell transcriptome. We report the first description of the transcriptome profiles of mouse bladder urothelial cells and highlight novel marker genes that can be used to categorize them. The transcription factor profiles of the bladder urothelium were also analysed and confirmed the heterogeneity of these eight subpopulations. The transcription factor profiles were significantly heterogeneous between basal and superficial subpopulations. In contrast to the previous studies which found two type of basal subpopulations (keratin‐5‐basal cells and keratin‐14‐basal cells), the present study found three basal‐like cell subpopulations with significantly different gene signatures. In addition, a unique cluster was also identified as the transitional state between basal and superficial cell. Moreover, three types of superficial‐like cell subpopulations were identified. Among these superficial‐like cell subpopulations, S2 was identified with the highest degree of cell differentiation. In particular, this analysis also revealed a previously uncharacterized population of superficial‐like cells labelled by plxna4. Interestingly, the present single‐cell RNA sequencing did not demonstrate all of the classic marker genes in the basal‐like cells. The extremely slow rate of urothelial turnover may be a factor that expression of marker gene(s) for stem or progenitor cells may be indistinguishable in normal bladder urothelium. The classification of bladder urothelial cells in the present study was also obviously different from that in the previous studies.^3^, ^31^ The obvious different classification method may account for this contradiction. With the technique of single‐cell RNA sequencing, the transcriptomic map of cell subpopulation rather than a limited number of markers can be analysed to define cell subtypes. In addition, the discrepancies between the present study and the previous studies as to the subtype of bladder urothelial cells could be due to the fact that the mouse bladder urothelium used in our study was in static state, rather than post‐injury. Microbial, chemical or processing/harvesting urothelial stimuli/injury can shift the bladder urothelium from near‐quiescence to a highly proliferative state, which can favour the lineage tracing study for the progenitor cells or stem cells. But it also broke the homeostasis of bladder urothelium and may impact the identities of bladder urothelial cells, which will provide the misleading information about the cell types. Harvesting and processing urothelial cells may also impact the transcriptomic profiles of the urothelial cells which could lead to spurious expression of genes.

As a crucial barrier between the blood and urine, the bladder urothelium has to recover quickly after injury. Conflicting evidence exists as to the location of progenitor cells responsible for urothelial restoration after injury. Previously, it is generally accepted that keratin‐5‐expressing basal cells are in charge of homeostatic maintenance and repair of the urothelium.^32^, ^33^ By utilizing a tamoxifen‐inducible *ShhCreERT2;mTmG* transgenic mouse, Shin et al found that sonic hedgehog (Shh)–expressing basal urothelial cells are capable of self‐renewal and differentiation.^4^ Other fate‐mapping studies demonstrated that superficial cells were derived from proliferation of both basal cells and intermediate cells after injury.^3^, ^34^, ^35^, ^36^ A more recent study showed that Wolffian duct epithelial cells can also repopulate injured bladders and restore a uroplakin barrier.^37^ These conflicting evidence might be the results of heterogeneity and complexity of bladder urothelial cells. In the present study, 8 subpopulations were identified according to the bladder urothelial cell transcriptome. Among these subpopulations, three subpopulations (B1, B2 and B3) are classified as basal‐like cells based on the marker genes. Developmental trajectory analysis and RNA velocity analysis suggested that B3 subpopulation are the progenitor cells that develop into B1 and B2. We further analyse the specific gene expressed in the B3 subpopulation and found that ASPM was the most highly expressed genes in B3 compared to other subpopulations. So we named this group as the ASPM^+^ urothelial cells. Genome‐wide analysis has suggested ASPM as a possible gastric stem/progenitor cell marker.^26^, ^27^ In the UTI model we established, we found that ASPM^+^ urothelial cells were significantly increased after acute injury, suggesting that ASPM^+^ urothelial cells are involved in the bladder urothelial regeneration. These evidence indicated that ASPM may also be a marker of progenitor cells in bladder urothelium. BTS subpopulation was regarded as the transitional state. We found that both BTS cells and I cells are responsible for the development of superficial cells (S1 and S2). These results are in line with previous lineage studies that both intermediate cells and basal cells can divide and produce superficial cells.^35^, ^38^

Bladder urothelial cells are closely related to the host response to UTIs and they are also a major source of proinflammatory cytokines and chemokines following bacterial infection.^39^, ^40^, ^41^ By using the scRNA‐seq of bladder urothelium, we discovered a novel bladder urothelial cell type characterized by the specific expression of Plxna4. IF results indicated that Plxna4^+^ urothelial cells were located on the apical layer of bladder urothelium in mouse, rat and human beings. Plexins are cell surface receptors that play an important role in axon guidance, cell migration, wound repair, mechanosensation, immune‐cell regulation and cancer progression.^42^, ^43^, ^44^ Until now, the expression and function of Plxna4 in bladder urothelium has not been reported. Wen et al demonstrate that Plxna4 is required for optimal cytokine production upon Toll‐like receptor (TLR) stimulation and bacterial challenge, suggesting a critical role of Plxna4 in mediating the host response to infection.^45^, ^46^ In addition, we found that Plxna4^+^ urothelial cells were highly enriched for WFDC1, which is a key modulator of inflammatory and wound repair responses.^47^ Hence, it is hypothesized that these Plxna4^+^ urothelial cells may play a role in the physiological process of host response to UTIs. It is hypothesized that these Plxna4^+^ superficial urothelial cells may act as ‘whistleblower cells’ that are involved in the initiation of host response during bacterial infection. Future studies on the function of plexna4^+^ urothelial cells are exigently worth researching. Additionally, these cells will provide a new insight into the bladder diseases associated with bladder urothelium.

Bladder cancers arise from the urothelial cells. Results of previous studies suggested that bladder cancers can be classified into distinct subtypes on the basis of morphology, gene expression, molecular changes and protein expression.^48^, ^49^, ^50^ Bladder cancer is determined by both genetic changes and the cell lineage from which the tumour originates.^51^ Different subtypes of bladder cancers may arise from distinct urothelial subpopulations. Van Batavia et al demonstrated that intermediate cells give rise primarily to papillary lesions, whereas Keratin‐5‐expressing basal cells are likely progenitors of carcinoma in situ, muscle‐invasive lesions and squamous cell carcinoma depending on its genetic background.^51^ Shin et al showed that muscle‐invasive bladder carcinomas arise exclusively from sonic hedgehog (Shh)–expressing basal cells.^52^ The other lineage tracing experiments indicated that keratin‐14‐expressing urothelial cells represent cells of origin of bladder cancer.^35^ Controversies about presence of progenitor (stem) cells giving rise to bladder cancer cells still exist among these studies. A possible explanation could be that the traditional classifications of bladder urothelial cells are not accurate. In the present study, we found a basal‐like cell population labelled by ASPM was the progenitor cell of the other subpopulations. Bioinformatic analysis based on TCGA and GEO Data found that high expression levels of ASPM are associated with the poor prognosis of patients with bladder cancer.^53^ In addition, ASPM is differentially expressed in patients with different races, weights, smoking habits and histological subtypes. These evidence suggested that bladder cancer arises from this urothelial cell population may be highly malignant and invasive. Future studies based on classification by single‐cell sequencing can help clarify clonal cell origin of bladder cancers, which will facilitate the development of targeted drug therapies.

Recent progress in single‐cell technologies has enabled the identification of all major cell types in mouse. It has been found that most cell types have distinct regulatory network structure and identifies regulators that are critical for cell identity.^18^ For urothelial cell types, the regulatory mechanisms underlying their identity remain poorly understood. The present study showed that bladder urothelial subpopulation had unique transcription factors, especially for the two newly discovered urothelial cell subpopulations (ASPM**^+^** and Plxna4**^+^** urothelial cells). Further analysis classified regulons into 4 major modules and M2 module contains 12 regulators, in which Foxa1, Grhl3, Foxq1, Grhl1, Htatip2, Ikzf2, Irf8 and Creb3l2 have been found to be mainly associated with cell differentiation.^21^, ^23^, ^54^, ^55^, ^56^, ^57^, ^58^, ^59^ It indicated that combinations of these regulons play important roles in urothelial cell differentiation. The analysis of regulon activity scores showing the activity of those regulons is more ‘active’ in Basal‐like cell subpopulations (cluster 0, 5, 6) than that in S2 cell subpopulations (cluster 4). This finding is consistent with the hypothesis that basal cell populations should have greater potential for growth and differentiation. Interestingly, at the gene expression level, the expression of all these 8 genes was more high in superficial cell subpopulations than other cell subpopulations. This contradiction can be explained by the fact that the analysis of the activity, rather than gene expression of transcription factors, may more accurately reflect its function.

BPS/IC has been described as a disease associated with impairment of bladder urothelium to regenerate. Many studies have described urothelial abnormalities as the predominant histopathologic findings in BPS/IC bladders. Since the bladder urothelium is comprised of several subpopulations with each subpopulation providing different functional roles, the significance of each subpopulation in the pathogenesis of BPS/IC need to be verified. However, studying the way by which specific urothelial subpopulation regulate bladder function is difficult. Our understanding of BPS/IC pathogenesis is limited by an incomplete molecular characterization of the cell types responsible for the impairment of bladder urothelial growth and differentiation. By comparing data from the scRNA‐seq of bladder urothelial cells, we sought to determine whether previously published IC/BPS‐associated genes are enriched for specific urothelial subpopulations. We found a number of IC/BPS‐associated genes differentially expressed among different urothelial subtypes. Especially, we found that most of IC/BPS‐associated genes are enriched in cluster 1, cluster 5 and cluster 6, which has basal cell‐like characteristics. The results suggested the cellular origin of IC/BPS might be these subpopulations of bladder urothelium or that the biopsies of the IC/BPS came for ulcerated areas of the bladder where superficial and intermediate cells have been denuded from the urothelium (denudation of the urothelium is a common finding in IC/BPS). Future studies using single‐cell sequencing to exploit cell type–specific changes during IC/BPS development are needed.

In the present study, the bladder urothelium of human beings was not used for the reason that it is impossible to get normal bladder urothelium from healthy volunteers. Since fresh cell suspensions are needed for the single‐cell RNA sequencing, bladder samples from the body donated take some time and this will greatly impact the cell activity and cell transcriptome. Another possible way to get the bladder urothelium is to use the bladder samples from cystectomy. However, patients undergoing cystectomies are usually diagnosed with muscle‐invasive bladder cancer. The bladder urothelium from these patients cannot be defined as normal bladder urothelium, especially if patients have been previously treated intravesical bacillus Calmette‐Guérin (BCG) or cytotoxic chemotherapy. Cystoscopic bladder urothelial biopsies could also be considered as another option to obtain normal human bladder urothelium. However, these biopsies are typically small and the number of urothelial cells obtained is low. These biopsies may lose some urothelial cell subpopulations, especially the basal cells. Hence, after careful deliberation, we decided to only perform the single‐cell sequencing from mouse bladder urothelial cells. Another limitation of this study is that there is no way to prove that these cluster profiles were not somehow contributed or affected by the way the cells were harvested/processed.

In conclusion, our study provides the most comprehensive information on the cell types of mouse bladders urothelium and opens up a range of possible avenues for future research. This work provides an important reference providing unprecedented and granular insights into the transcriptional landscape of bladder urothelial cells. This information is fundamental to understanding bladder biology and bladder diseases.

## CONFLICT OF INTEREST

The authors declare that they have no conflict of interest.

## AUTHOR CONTRIBUTIONS

Dr Li and Dr Liu (1) performed scRNA‐seq experiments and analyses, performed HE stain and immunofluorescence, made figures and wrote the paper; Dr Gao, Dr Zhang and Dr Chen dissected mouse bladder urothelium, performed RNA‐seq experiments and wrote the paper; Dr Wu and Dr Liu (2) dissected mouse bladder urothelium and made UTIs models; Dr Wang and Mr Zhou performed bioinformatics; Prof. Chai conceived of the project and critically reviewed the manuscript; Prof. Shi supervised the project and wrote the paper; and all authors read and commented on the manuscript.

## Supporting information

FigS1

FigS2

FigS3

FigS4

FigS5

Supplementary Material

## Data Availability

The data that support the findings of this study are available in GEO datasets at [https://www.ncbi.nlm.nih.gov/gds/?term=Single+cell+transcriptomes+of+mouse+bladder+urothelium+uncover+novel+cell+type+markers+and+urothelial+differentiation+characteristics]. Series Accession: GSE163029. ID: 200 163 029.
