# Supplementary figures and images for: Single‐cell transcriptomes of mouse bladder urothelium uncover novel cell type markers and urothelial differentiation characteristics
Source: Cell Prolif. 2021 Feb 3;54(4):e13007. doi: 10.1111/cpr.13007 (PMC8016651; doi:10.1111/cpr.13007)

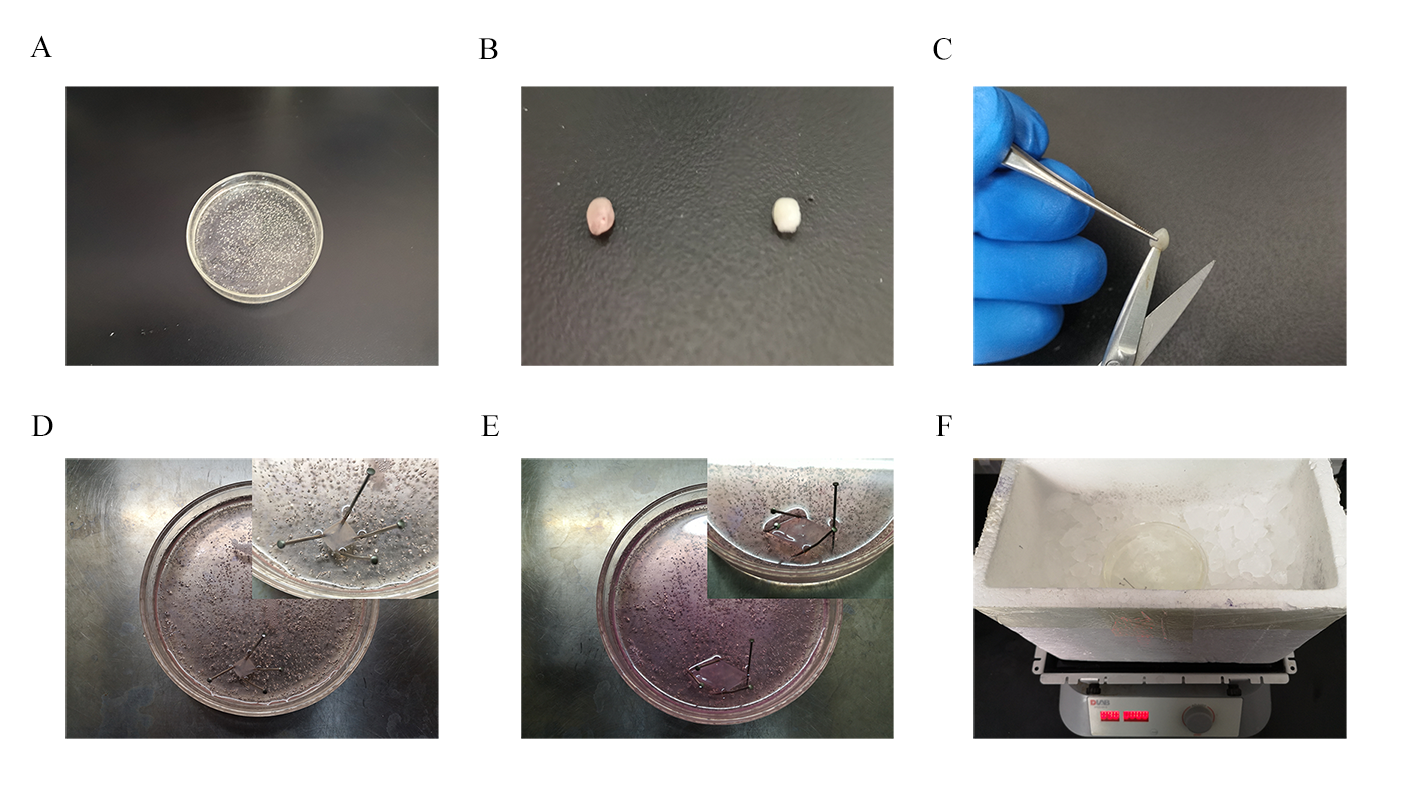

Supplement: Supplementary file 1 — FigS1 [file CPR-54-e13007-s004.tif]

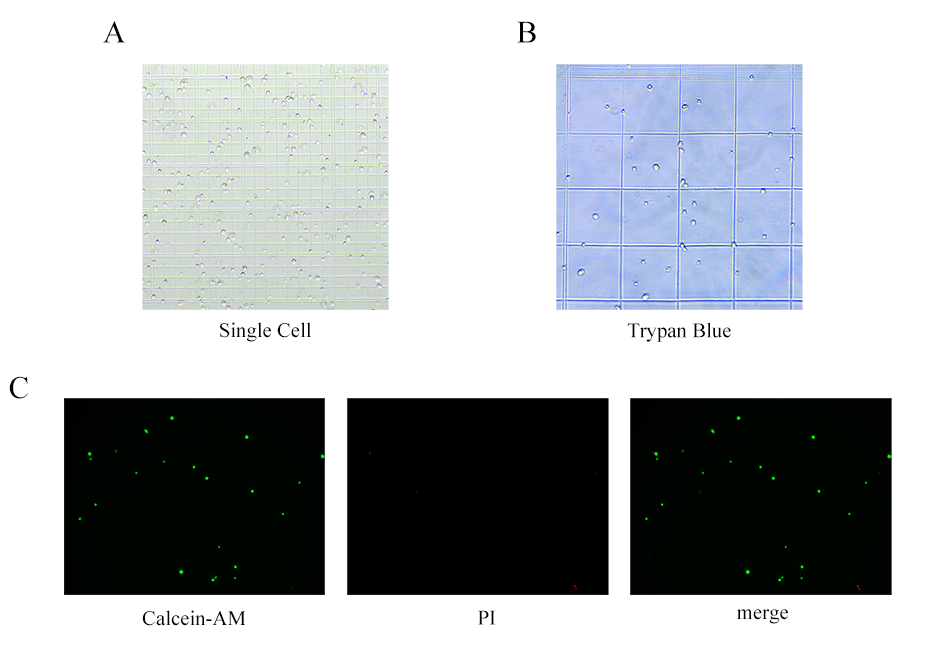

Supplement: Supplementary file 2 — FigS2 [file CPR-54-e13007-s006.tif]

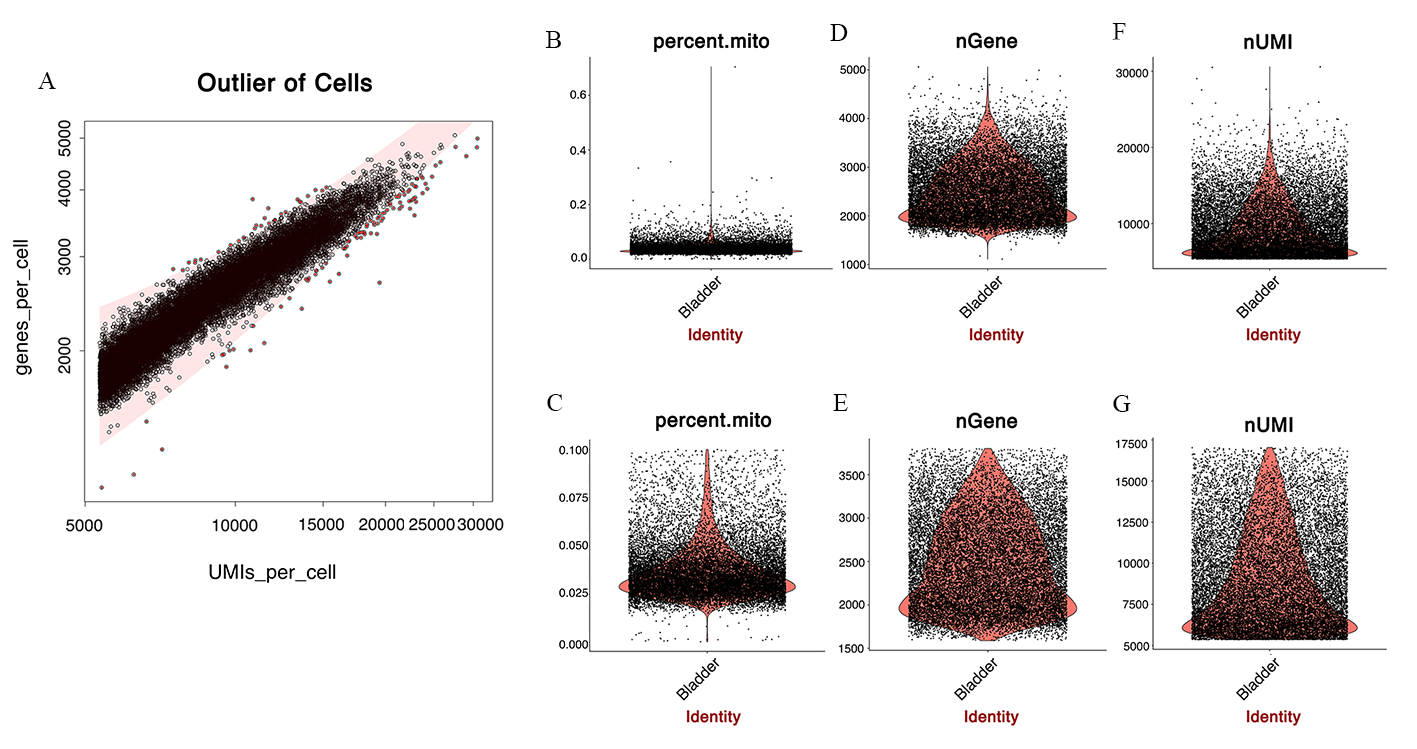

Supplement: Supplementary file 3 — FigS3 [file CPR-54-e13007-s005.tif]

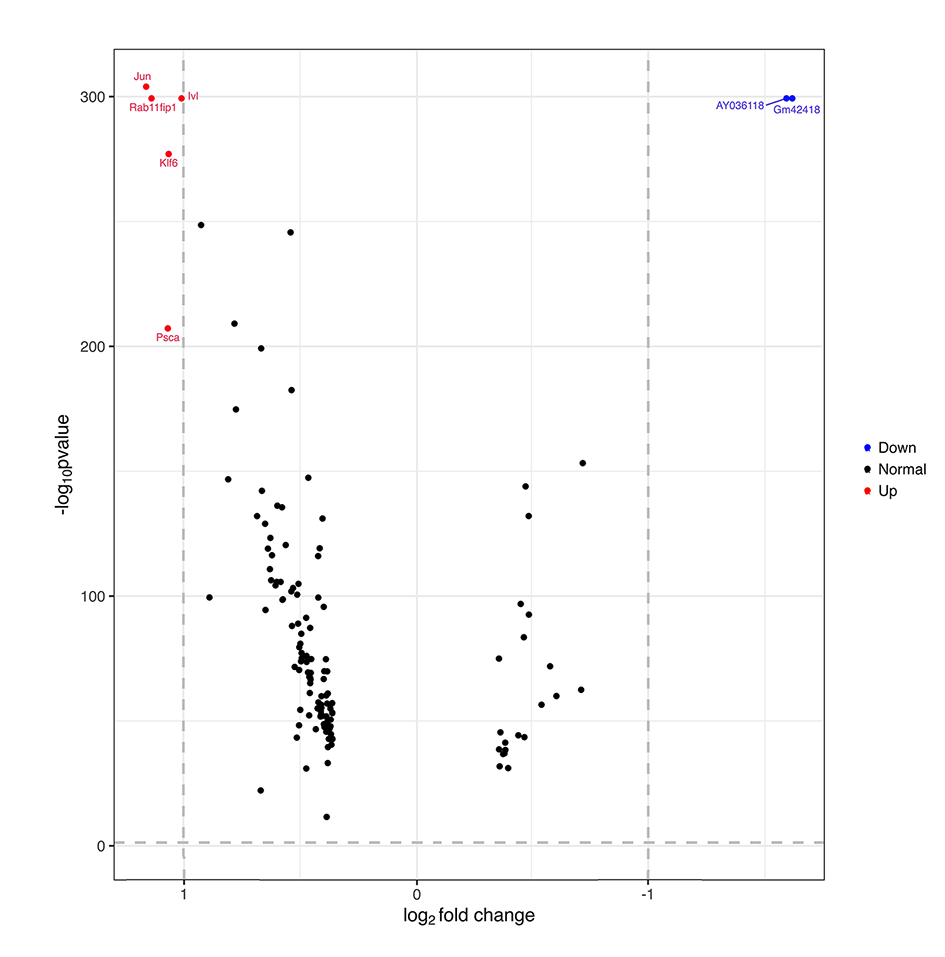

Supplement: Supplementary file 4 — FigS4 [file CPR-54-e13007-s001.tif]

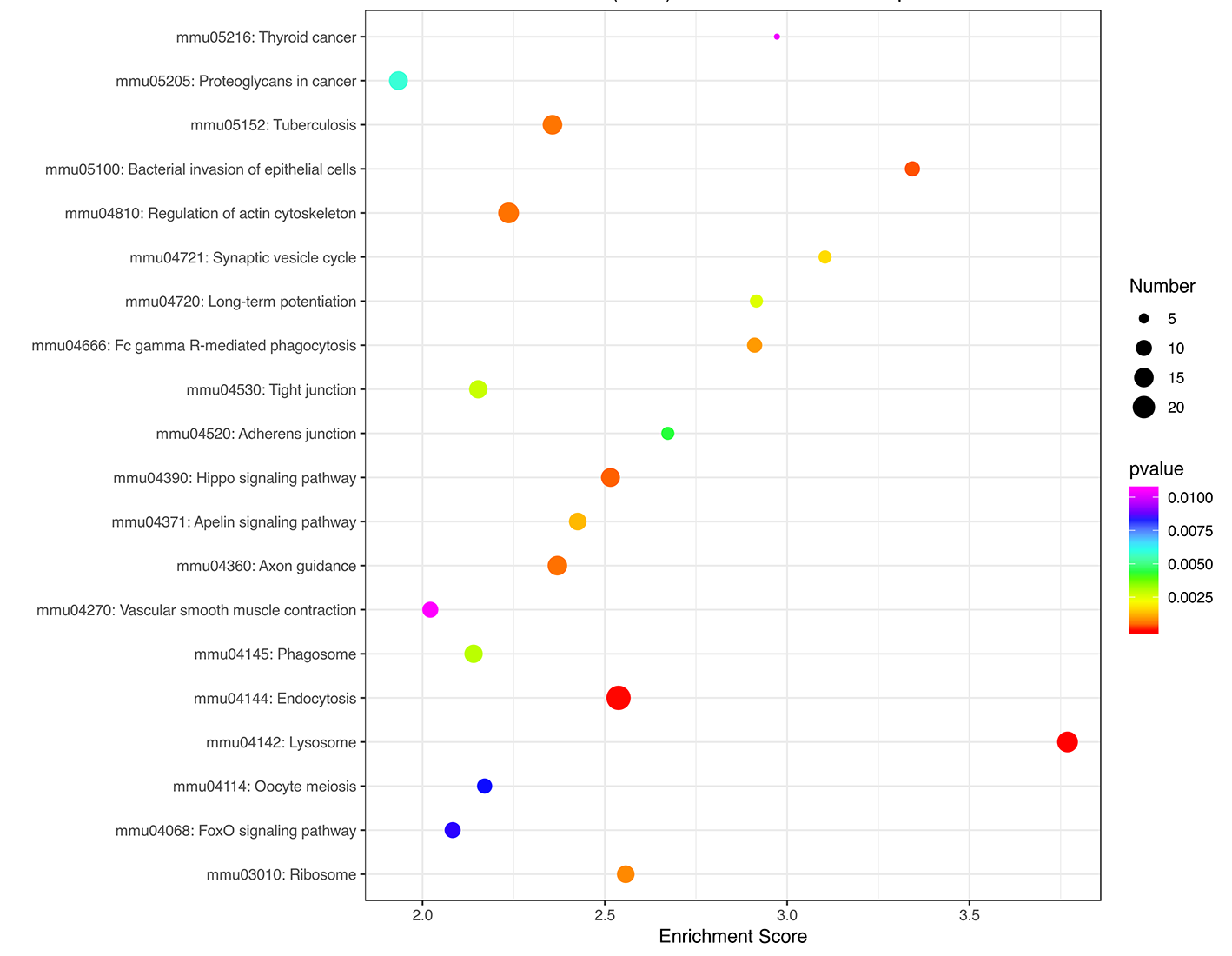

Supplement: Supplementary file 5 — FigS5 [file CPR-54-e13007-s002.tif]
