# Supplementary material for: Single‐cell transcriptomes of mouse bladder urothelium uncover novel cell type markers and urothelial differentiation characteristics
Source: Cell Prolif. 2021 Feb 3;54(4):e13007. doi: 10.1111/cpr.13007 (PMC8016651; doi:10.1111/cpr.13007)
Supplement: Supplementary file 6 — Supplementary Material [file CPR-54-e13007-s003.docx]

**Supplementary Figure 1. Key points of bladder urothelial cell Isolation.**

(A) The Petri dish is filled with the 30-40ml silicone elastomer. Using 75% (vol/vol) ethanol to soak the surgical instruments, pins and Sylgard-coated plate for at least 30min, and then wash them by [sterile](javascript:;) [water](javascript:;) and airing in the super clean bench. This step needs to be performed 30 minutes before harvesting bladders.

(B) After [myocardial](javascript:;) [perfusion](javascript:;), cut off the bladder at the bladder neck with the sharp scissors and put it on the Sylgard-coated plate. The pale coloured bladder is the sign of Perfusion success (Right).

(C) Expose lumen of the bladder by inserting one arm of a scissors through the small opening created when dissecting the bladder.

(D) Pin bladder flat near the corner of the plate making sure lumen side is up. Use other three pins pin bladder flat to form a parallelogram. Rinse bladder with 3mL DMEM and aspirate to wash away any contained urine or attached hair. Repeat two more times.

(E) Incubate bladder at 37°C for 1.5 hours in dispase II solution. So we could make bladder achieve a maximum stretch.

(F) Poor 10-15ml PBS-EDTA in Sylgard-coated plate to incubate bladder. Then put Sylgard-coated plate on the ice, gentle shaking for 1.5 hours. Collect PBS-EDTA into 15ml centrifuge tube. Cells were then digested in 1ml TrypLE™ Express Enzyme for 1min and were neutralized by 10ml 0.04%BSA D-PBS.

**Supplementary Figure 2. Cell morphology and activity.**

1. The number of cells were counted by the blood cell counting plate. The activities were tested by Trypan blue stain (B) and Calcein-AM/PI stain (C).

**Supplementary Figure 3. Quality control of single cell data.**

1. The urothelial cells were filtered by fitting the generalized linear model. Ratio distribution of mitochondrial gene transcription in single cell before (B) and after (C) quality control. Distribution of the number of genes expressed in individual cells before (D) and after (E) quality control. Distribution of the number of UMI in a single cell before (F) and after (G) quality control.

**Supplementary Figure 4. Volcano Plots showing** [**differential**](javascript:;) [**gene**](javascript:;)**s between cluster 3 and cluster 4.**

**Supplementary Figure 5. Target genes of Foxa1, Grhl3, Foxq1, Grhl1,** **Htatip2,** **Ikzf2, Irf8 and Creb3l2 were searched by KEGG pathways database which are enriched for epidermal barrier formation, such as regulation of actin cytoskeleton, bacterial invasion of epithelial cells, endocytosis and lysosome.**
